# Human Small Airway Epithelia Reveal Dichloroacetate as a Broad-Spectrum Antiviral Against Respiratory Viruses

**DOI:** 10.3390/ijms26209853

**Published:** 2025-10-10

**Authors:** Paula Martínez de Iturrate, Bruno Hernáez, Patricia de los Santos, Yolanda Sierra-Palomares, Alba García-Gómez, Alonso Sánchez-Cruz, Catalina Hernández-Sánchez, Luis Rivas, Margarita del Val, Eduardo Rial

**Affiliations:** 1 Centro de Investigaciones Biológicas Margarita Salas, CSIC, 28040 Madrid, Spain; 2 Centro de Biología Molecular Severo Ochoa, CSIC-UAM, 28049 Madrid, Spain; 3 Centro de Investigación Biomédica en Red de Diabetes y Enfermedades Metabólicas Asociadas (CIBERDEM-ISCIII), 28034 Madrid, Spain

**Keywords:** coronavirus, respiratory syncytial virus, respiratory infection, small airway epithelia, energy metabolism, dichloroacetate, antiviral

## Abstract

Respiratory viral infections are a major cause of morbidity and mortality worldwide. The COVID-19 pandemic has evidenced the need for broad-spectrum antivirals and improved preclinical models that more accurately recapitulate human respiratory disease. These new strategies should also involve the search for drug targets in the infected cell that hamper the development of resistance and of potential efficacy against diverse viruses. Since many viruses reprogram cellular metabolism to support viral replication, we performed a comparative analysis of inhibitors targeting the PI3K/AKT/mTOR pathway, central to virus-induced metabolic adaptations, using MRC5 lung fibroblasts and Huh7 hepatoma cells. HCoV-229E infection in MRC5 cells caused the expected shift in the energy metabolism but the inhibitors had markedly different effects on the metabolic profile and antiviral activity in these two cell lines. Dichloroacetate (DCA), a clinically approved inhibitor of aerobic glycolysis, showed antiviral activity against HCoV-229E in MRC5 cells, but not in Huh7 cells, underscoring that the screening model is more critical than previously assumed. We further tested DCA in polarized human small airway epithelial cells cultured in air–liquid interface, a 3D model that mimics the human respiratory tract. DCA reduced the viral progeny of HCoV-229E, SARS-CoV-2, and respiratory syncytial virus by 2–3 orders of magnitude, even when administered after infection was established. Our work reinforces the need for advanced human preclinical screening models to identify antivirals that target host metabolic pathways frequently hijacked by respiratory viruses, and establishes DCA as a proof-of-concept candidate.

## 1. Introduction

Respiratory viruses are among the leading causes of morbidity and mortality worldwide. Influenced by human density and mobility, and climate change, these viruses have been responsible for a variety of outbreaks and epidemics with important social and economic consequences [1]. This has been the case for the severe acute respiratory syndrome (SARS) outbreak in 2003, influenza H1N1 in 2009, Middle East respiratory syndrome (MERS) in 2012 and, most recently, the COVID-19 pandemic. Prevention or containment of these emerging and re-emerging respiratory viral diseases requires the development of novel antiviral strategies and prophylactic vaccines. Unfortunately, during the COVID-19 pandemic, standard antiviral screening protocols achieved only limited success in developing novel human therapies [2,3], underscoring the lack of chemotherapeutic tools to combat emerging viruses.

The antiviral activity of drugs is often evaluated using immortalized cell lines. However, when dealing with respiratory viruses, these high-throughput screening platforms may not reproduce conditions that could be relevant to the progression of the infection, such as the presence of ciliated or mucus-secreting cells [2,3,4,5,6,7]. Moreover, most approaches designed to obtain antiviral agents have targeted the virus, either on its entry into the host cell or its replication within the cell. These include developing antibodies or small competitive peptides to prevent the virus from binding to the cell’s receptors or inhibitors of viral proteases or replicases [8,9,10]. While such approaches may be effective, their long-term therapeutic applications could suffer from two major drawbacks: (i) the appearance of new resistant strains that evolve to render these antivirals ineffective is possible and (ii) the agents developed may be active against only a small group of viruses and not effective against new viral strains or emerging viral diseases. However, targeting cellular pathways that viruses commonly hijack and use to replicate could overcome these problems [11].

Viruses use the metabolic machinery of infected cells to synthesize the components needed to generate viral progeny. This process involves the reprogramming of cellular metabolism to provide the metabolic precursors, which generally includes an increased activity of pathways such as glycolysis, nucleotide biosynthesis or glutaminolysis [12,13,14,15]. However, the biological adaptations required are context-dependent, and thus the metabolism of tumor cells provides a different background to the infecting virus than that of a terminally differentiated cell [16]. Therefore, the efficiency of the replication cycle is influenced by both its intrinsic characteristics and the physiological context within the infected cell. Slow-replicating viruses must adopt strategies to avoid cell lysis induction by maintaining a permissive metabolic environment and actively preventing apoptosis to gain time for virion assembly. In contrast, fast-replicating viruses can cope with cellular dysfunction and actively trigger programmed cell death as a dissemination strategy [17,18,19].

The term “Warburg effect” is used to describe a cellular metabolic state with an increased glucose uptake, glycolysis, and lactate secretion, despite the presence of oxygen. The Warburg effect was initially defined in the context of cancer cells, although it reflects the rewiring of metabolism when cells respond to enhanced demands to synthesize cellular components (proteins, lipids, DNA, etc.), either to maintain a proliferative state [20] or, as in the case of virus-infected cells, to produce viral progeny [13,14,21]. The signaling pathways that govern this anabolic state have been well characterized [21,22,23,24], and one of the most prominent pathways is the signaling network PI3K/AKT/mTOR (growth factor-regulated phosphoinositide 3-kinase—serine/threonine kinase Akt—mammalian target of rapamycin). Notably, hypoxia inducible factor 1 (HIF-1α) is downstream of the PI3K/AKT/mTOR pathway and is a major regulator of energy metabolism.

Over the last decade, an increasing number of publications have focused on characterization of the virus-induced metabolic reprogramming of host cells [12,13,14,15,21,22,24,25,26,27] with the aim of identifying targets that may lead to new approaches for therapeutic intervention [24,25,26,27,28]. Since the pathways involved in the Warburg rewiring of cancer cells have been the subject of detailed analyses to develop anti-tumor therapies [29,30], it is likely that some of the available drugs could also be used as antivirals. Indeed, some of the inhibitors of glycolysis, glutaminolysis or fatty acid synthesis that are considered to be anticancer agents also display activity against different viruses [11,25]. However, their use in the treatment of respiratory viral infections has not yet been studied.

Here, we investigated the antiviral activity of drugs targeting the PI3K/AKT/mTOR pathway in MRC5 lung fibroblasts and the hepatoma Huh7 cell line, both infected with the HCoV-229E coronavirus. Surprisingly, we found that the pyruvate dehydrogenase kinase (PDK) inhibitor dichloroacetate (DCA) has antiviral effects on MRC5-infected but not on Huh7-infected cells. The inhibitor was then tested in a more appropriate system for respiratory viruses, polarized human small airway epithelia (hSAEC) in air-liquid interface, and the results show that DCA markedly reduced the progeny of three human respiratory viruses—the coronaviruses HCoV-229E, SARS-CoV-2 and the respiratory syncytial virus (RSV)—even if applied when the infection is established.

## 2. Results

### 2.1. Viral Infection Leads to Metabolic Rewiring in MRC5 Fibroblasts

**Figure 1:**
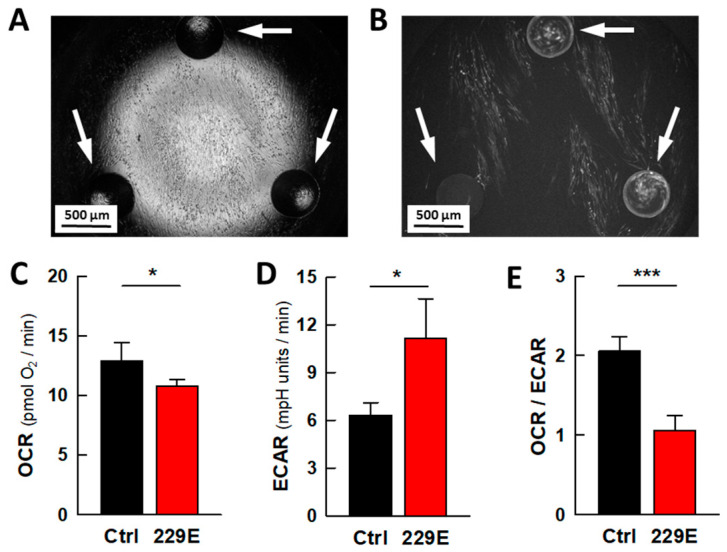
Coronavirus infection alters the energy metabolism of MRC5 cells. (**A**,**B**) Representative images of MRC5 cells cultured in an XFp microwell plate and infected with the coronavirus HCoV-229E. The arrows point to the circular well bumps at the bottom of the plate that define the height of the microchamber. (**A**) Bright field microscopy image of MRC5 cells seeded in an XFp microwell. (**B**) Fluorescence microscopy image showing cells infected with the GFP-labeled virus HCoV-229E in the XFp well. (**C**–**E**) Effect of viral infection on cell bioenergetics. (**C**) Rate of respiration (OCR), (**D**) aerobic glycolysis (ECAR), and (**E**) metabolic profile (the OCR/ECAR ratio). The data are presented as the mean ± s.e.m. of 4 biologically independent experiments performed in triplicate. The statistical significance of the differences was calculated via an unpaired Student’s *t* test. * *p* < 0.05; *** *p* < 0.001.

The energy metabolism of MRC5 lung fibroblasts was assessed using an XFp extracellular flux analyzer to determine whether the metabolic profile of these cells is altered when they are infected by the common cold coronavirus HCoV-229E (Figure 1). The analysis was conducted in the absence of heat-inactivated fetal bovine serum (HIFBS), as MRC5 cells are proliferative and therefore have a Warburg metabolism. Since serum removal should result in cell growth arrest, it allows the metabolic effect of the infection to be better defined. Therefore, infections were carried out in medium containing HIFBS, as indicated in Section 4, and the bioenergetic analysis was conducted in the absence of HIFBS 48 h later. The change to the serum-deprived Dulbecco’s modified Eagle’s medium (DMEM) was carried out one hour prior to the metabolic analysis. Under these conditions, infected cells exhibited a remarkably higher rate of glycolysis (extracellular acidification rate, ECAR) accompanied by a minor decrease in respiration (oxygen consumption rate, OCR). These combined effects are indicative of a viral-induced metabolic rewiring, clearly reflected in the OCR/ECAR ratio (Figure 1E).

### 2.2. Antiviral Effects of Inhibitors of Pathways Involved in the Warburg Effect

**Figure 2:**
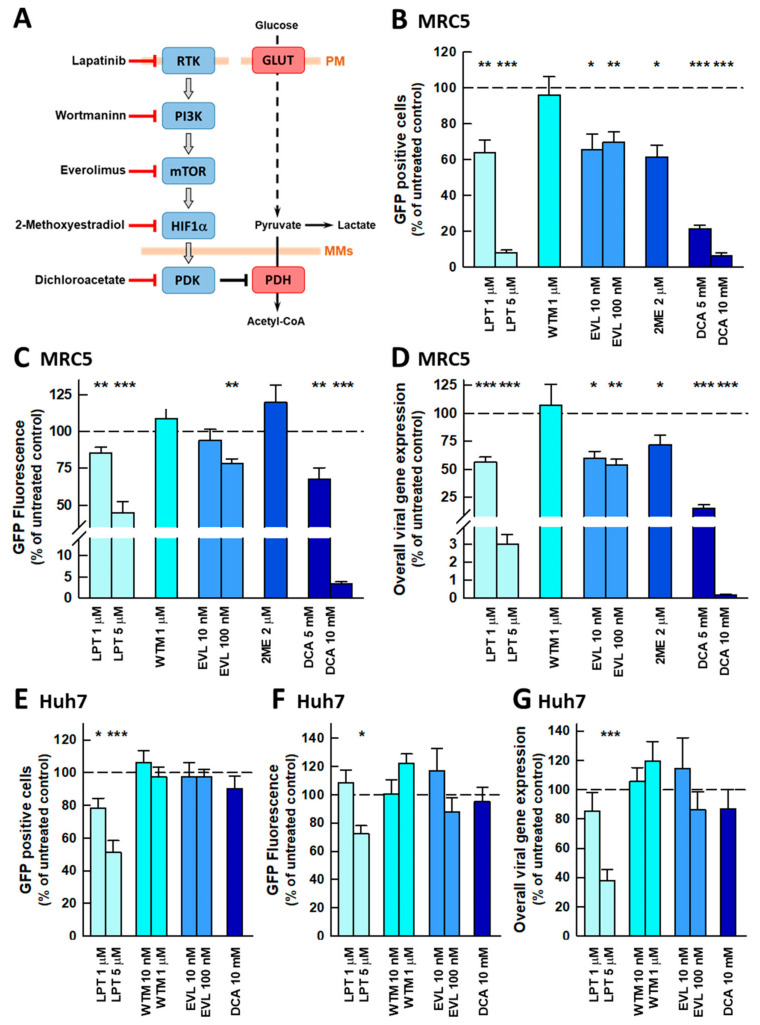
The antiviral activity of PI3K/AKT/mTOR signaling pathway inhibitors in MRC5 cells infected with HCoV-229E. (**A**) Scheme of the signaling pathway and the inhibitors whose antiviral activity was studied. Effect of the pathway inhibitors on the progression of infection in the lung fibroblast cell line MRC5 (**B**–**D**) or the hepatoma cell line Huh7 (**E**–**G**); (**B**,**E**) percentage of infected cells; (**C**,**F**) mean GFP fluorescence of the infected cells; (**D**,**G**) overall virus gene expression calculated from the percentage of infected cells and the mean fluorescence intensity. The bars represent the mean ± s.e.m. values, and the number of biologically independent experiments ranged from 6 to 12 (MRC5) or 5 to 7 (Huh7); all the experiments were performed in triplicate. The statistical significance of the differences was calculated using an unpaired Student’s *t* test relative to the untreated control. * *p* < 0.05; ** *p* < 0.01; *** *p* < 0.001. Where not indicated, the differences were not significant. Abbreviations: PM, plasma membrane; MMs, mitochondrial membranes; LPT, lapatinib; WTM, wortmaninn; EVL, everolimus; 2ME, 2-methoxyestradiol; DCA, dichloroacetate.

To determine whether the PI3K/AKT/mTOR signaling cascade is involved in the observed viral-induced metabolic reprogramming, we selected a set of inhibitors that target different elements in this signaling pathway (Figure 2A). Specifically, we assessed the following: lapatinib (LPT), which inhibits the epidermal growth factor receptors EGFR and HER2 tyrosine kinases; wortmaninn (WTM), an irreversible inhibitor of class I and III PI3Ks; everolimus (EVL), which inhibits the mTORC1 complex through interaction with FK-binding protein 12 (FKBP12); 2-methoxyestradiol (2ME), an inhibitor of HIF-1α mRNA translation; and DCA which inhibits PDKs to release the inhibition of pyruvate dehydrogenase (PDH) and allow the mitochondrial oxidation of pyruvate. Each inhibitor was used at a concentration commonly used in the literature and always above the reported IC_50_ values (see Table S1). DCA was used at 1–10 mM that are the in vitro concentrations generally used to mimic the cytosolic levels of pyruvate that inhibit PDK [31].

The effects of these inhibitors on viral infection were assessed (Figure 2B–D), after adjusting the adsorption time and duration of infection to minimize cell lysis, thereby allowing quantification of the effect of the selected drugs on the progression of viral infection. Three parameters were used to evaluate the antiviral activity of the inhibitors: the percentage of infected cells (Figure 2B), the mean viral-driven GFP fluorescence in the infected cells (Figure 2C), and overall viral gene expression (Figure 2D). This latter parameter was derived from the multiplication of the two other parameters and acted as an indirect indicator of the progression of viral infection. Among the untreated cells, the proportion of infected cells at 72 hpi (hours post-infection) was 19.0 ± 2.3 (*n* = 38). As expected, all the inhibitors tested, except WTM, produced a significant decrease in overall viral gene expression, although the strongest effects were observed with LPT (5 μM, 97% ± 3) and DCA (10 mM, 99.97% ± 0.01).

Primary drug screening, particularly antiviral screening, is often performed using tumor cell lines because of their low maintenance costs and their adaptability to high-throughput approaches. Although tumor cells already undergo Warburg metabolism, for comparison, the inhibitors were tested in Huh7 hepatoma cells, a cell line widely used to search for new antiviral drugs [32,33]. As with MRC5 cells, infection conditions were adjusted to minimize the higher lytic activity of the virus in the hepatoma cell line. Thus, the percentage of infected cells at 24 hpi was 14.6 ± 0.5 (*n* = 14), and under these conditions, only LPT caused a significant decrease in the viral load (Figure 2E–G). Strikingly, DCA produced only a marginal effect.

**Figure 3:**
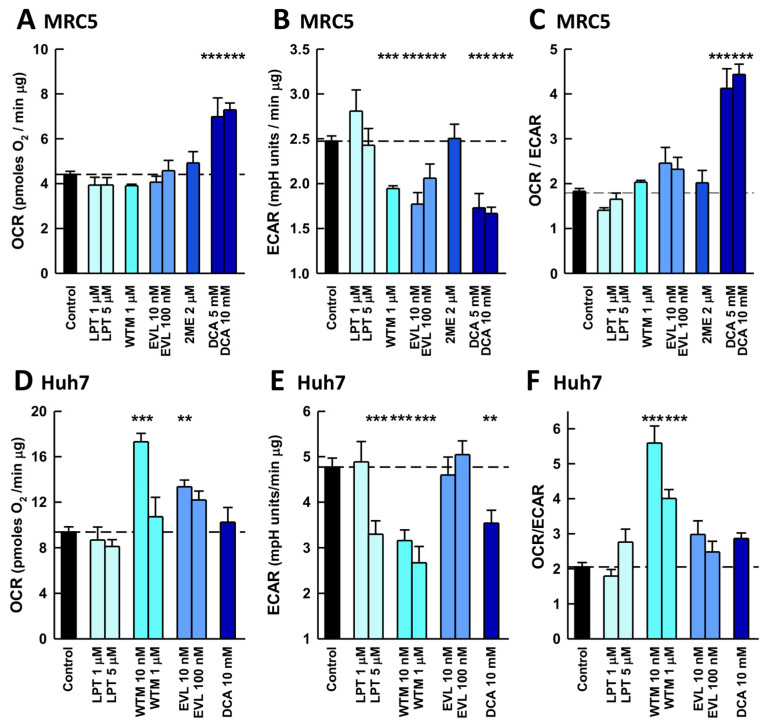
Inhibitors of the PI3K/AKT/mTOR signaling pathway differentially alter the metabolism of uninfected MRC5 and Huh7 cells. The effect of PI3K/AKT/mTOR pathway inhibitors on the energy metabolism of MRC5 (**A**–**C**) and Huh7 (**D**–**F**) cells: (**A**,**D**) Rate of respiration (OCR), (**B**,**E**) aerobic glycolysis (ECAR), and (**C**,**F**) metabolic profile (OCR/ECAR ratio). The data are presented as the mean ± s.e.m. of 5–14 biologically independent experiments performed with 5 technical replicates. The statistical significance of the differences was calculated using an unpaired Student’s *t* test relative to the untreated control. ** *p* < 0.01; *** *p* < 0.001. Where not indicated, the differences were not significant. Abbreviations: LPT, lapatinib; WTM, wortmaninn; EVL, everolimus; 2ME, 2-methoxyestradiol; DCA, dichloroacetate. Dashed line indicates control value in every case.

The observed differences in the antiviral activity of the pathway inhibitors between these two distinct cell lines pointed to disparities in their respective signaling networks and, therefore, the effects of the inhibitors on the energy metabolism of uninfected cells were investigated. EVL, WTM, and DCA caused significant decreases in glycolysis in MRC5 lung fibroblasts, but only DCA induced a compensatory increase in respiration (Figure 3A–C). Therefore, the OCR/ECAR analysis identified DCA as the sole compound to cause a metabolic shift consistent with the inhibition of the Warburg effect, and consequently, its impact on MRC5 bioenergetics was characterized further (Figure S1). The responses of Huh7 hepatoma cells differed in response to the same set of inhibitors (Figure 3D–F). Low concentrations of WTM (10 nM) strongly inhibited the Warburg effect, whereas DCA had only a small effect on glycolysis without a compensatory increase in the OCR. Comparing the effects of these inhibitors on cell bioenergetics with their antiviral activity does not provide a clear pattern. Except for WTM, the inhibitors with antiviral activity also inhibited glycolysis in MRC5 fibroblasts. This finding was in sharp contrast to the effects in Huh7 cells where, despite inhibiting glycolysis, WTM and DCA did not display antiviral activity. Intriguingly, LPT produced antiviral activity in both cell types but without significantly altering their metabolism. Remarkably, DCA is the drug that causes the strongest inhibition of the Warburg effect in MRC5 cells and has the strongest antiviral activity.

### 2.3. Antiviral Activity of DCA in Small Airway Epithelia

**Figure 4:**
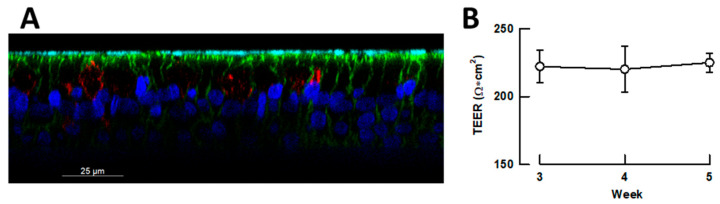
Characterization of the polarized human small airway pseudo-stratified epithelia used in the studies. (**A**) Representative confocal fluorescence microscopy image of the morphology of the small airway epithelia. Image show nuclei stained with DAPI (blue), α-tubulin-labeled cilia (cyan), F-actin stained with phalloidin (green) and MUC5AC expressing goblet cells (red). (**B**) Barrier integrity of the differentiated epithelia assessed by the trans-epithelial electrical resistance (TEER). The data points represent the means ± s.e.m. of 3–5 independent differentiation plates, averaging measurements from 6 randomly selected inserts in each.

There is a growing consensus on the need for appropriate experimental models for preclinical drug characterization [34,35,36,37,38]. The use of polarized small airway epithelia in air-liquid interface cultures (ALI) is likely to better mimic the physiological context of respiratory virus infection, providing a better setting in which to test new antiviral drugs. Thus, we studied the in vitro infection of small airway epithelia with the coronaviruses HCoV-229E and SARS-CoV-2, and the pneumovirus RSV. Confocal microscopy of 4′,6-diamidino-2-phenylindole (DAPI) stained preparations revealed a pseudostratified epithelium with multiple rows of nuclei (Figure 4A). Cilia projections, identified by α-tubulin staining, were restricted to the apical surface, confirming epithelial polarization. Secretory cells were detected by immunostaining with an anti-MUC5AC antibody, and F-actin, visualized with phalloidin, delineated the borders of individual epithelial cells (Figure 4A and Figure S2A–D). Transepithelial electrical resistance (TEER) measurements obtained during weeks 3–5 of differentiation corroborated the presence of a well-formed, pseudostratified, polarized airway epithelium (Figure 4B).

**Figure 5:**
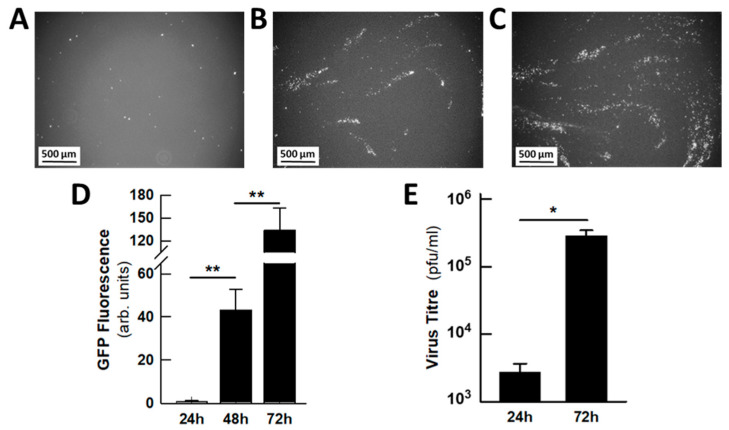
Progression of HCoV-229E infection in human pseudostratified small airway epithelia. (**A**–**C**) Representative fluorescence microscopy images of the spiral progression of infection at (**A**) 24, (**B**) 48 and (**C**) 72 hpi. (**D**) Quantification of the GFP fluorescence. (**E**) Viral titre in the mucus recovered from the apical phase of the epithelia at 24 and 72 hpi. The number of inserts (epithelia) evaluated in (**D**) were 14 (control), 6 (24 hpi), 37 (48 hpi) and 37 (72 hpi). For (**E**), the number of inserts was 5 (24 hpi) and 14 (72 hpi). The bars represent the mean ± s.e.m. of those determinations, and the significance of the differences was calculated using one-way ANOVA (**D**) or an unpaired Student’s *t* test (**E**). * *p* < 0.05; ** *p* < 0.01.

We first monitored the progression of HCoV-229E-GFP infection from the apical side of the epithelia under the fluorescence microscope. Images at 24 h intervals revealed that the viral infection spreads in a spiral mode, probably as a result of cilia movement (Figure 5A–C). Fluorescence quantitation showed the gradual increase in GFP expression as viral infection progressed (Figure 5D), which was concordant with the increase in viral titre from 24 to 72 hpi (Figure 5E). Indeed, the production of infectious viral particles in the mucus increased by two orders of magnitude during this period. Since no infectious viruses were detected in the basolateral compartment, the amount of virus present in the mucus represents the total amount of virus released. During the routine maintenance of the epithelia, the mucus was washed off every two days; however, during infection, the mucus was only removed at the end of the experiment and used for viral titration.

**Figure 6:**
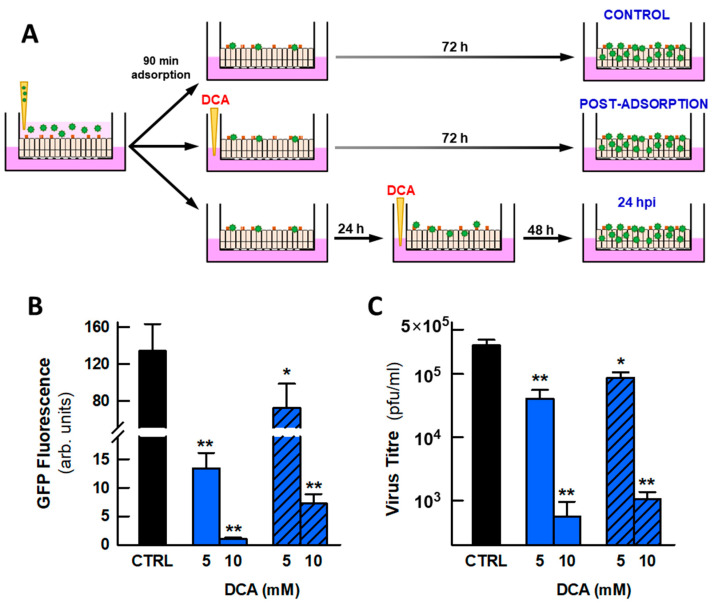
DCA inhibits the progression of HCoV-229E infection in human pseudostratified small airway epithelia. (**A**) Scheme of the protocol used to investigate the antiviral activity of DCA. The drug was added to the basolateral compartment either immediately after virus adsorption or 24 h later. The viral content was determined in both cases at 72 hpi. (**B**) Effect of DCA on the virus-driven GFP fluorescence in the epithelia. (**C**) Virus titre in the mucus recovered from the apical phase of the epithelia at 72 hpi. The hatched bars correspond to drug treatments initiated at 24 hpi. The number of inserts (epithelia) evaluated was 20–30 for each condition in (**A**) and 8–14 for (**B**). The bars represent the means ± s.e.m. values, and the significance of the differences was calculated using an unpaired Student’s *t* test for each condition relative to the control. * *p* < 0.05; ** *p* < 0.01.

To test the antiviral activity of DCA in this system, two protocols were used (Figure 6A): one that commenced by adding DCA to the basolateral medium immediately after viral adsorption, and the other to test the effect of the drug once viral infection was established by adding DCA to the basolateral medium at 24 hpi. In both cases, infection was allowed to proceed until 72 hpi and when the effects of DCA were quantified. The addition of DCA (10 mM) to the basolateral compartment decreased viral titres by two orders of magnitude, almost completely abolishing viral replication, as assessed through GFP fluorescence and the virus titre in the apical mucus (Figure 6B,C). Notably, DCA had a clear impact even when added after infection had been established and, furthermore, the viral titre at 72 hpi was lower than the titre at the time of drug addition (24 hpi). Indeed, the viral load detected in the mucus prior to DCA addition was close to 2700 pfu/mL, whereas it had fallen to 1040 ± 320 pfu/mL 48 h later. This reduction in viral titre suggests that DCA not only dampens the progression of viral infection but also contributes to the clearance of HCoV-229E viruses from the epithelia. As LPT has demonstrated antiviral activity in MRC5 and Huh7 cells and it has been reported to have antiviral activity against SARS-CoV-2 in MRC5 cells [39], its effects on HCoV-229E-infected epithelia were also tested. However, there was no evidence that it had any statistically significant effect (Figure S3A,B).

**Figure 7:**
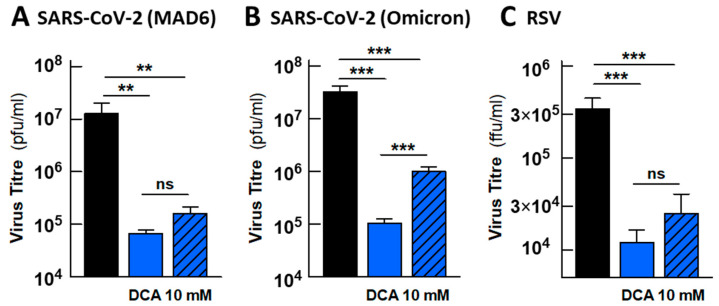
DCA inhibits the progression of SARS-CoV-2 and RSV infection in human pseudostratified small airway epithelia. Effect of DCA on infection by the SARS-CoV-2 strains MAD6 (**A**) and Omicron (**B**), or RSV (**C**). The virus titre in the mucus recovered from the apical phase of the epithelia was measured at 72 hpi. The hatched bars correspond to drug treatments initiated at 24 hpi. There were 6 inserts (epithelia) evaluated for each condition in (**A**), 11–17 for (**B**) and 12–16 for (**C**). The bars represent the means ± s.e.m. values, and the significance of the differences between the indicated pairs of conditions was calculated using an unpaired Student’s *t* test. ** *p* < 0.01; *** *p* < 0.001; ns, not significant difference.

We subsequently tested in the small airway epithelia the antiviral effect of DCA against two additional respiratory viruses with a high incidence in human health: the coronavirus SARS-CoV-2 (strains MAD6 and Omicron) and the RSV A pneumovirus. It has already been shown that cells freshly isolated from the upper and lower airways of pediatric patients with RSV infection display an enhanced glycolytic metabolism [28]. DCA exhibited a marked antiviral effect on both viruses, decreasing the viral titres by more than two orders of magnitude in SARS-CoV-2-infected epithelia and only slightly less for RSV. The drug also provided a significant protective effect when added when the infection was established (24 hpi), as observed with HCoV-229E (Figure 7). The data obtained with RSV in the epithelia prompted us to test the effect of DCA in RSV-infected MRC5 cells. RSV has only mild lytic activity in this cell model and therefore, it was possible to reach a high proportion of infected cells at 72 hpi (73.6 ± 3.0%, *n* = 8). However, DCA added after viral adsorption had relatively weak antiviral activity, although it did provoke a significant decrease in overall viral gene expression (49%, Figure 7C).

## 3. Discussion

A variety of virus families are responsible for respiratory viral infections, which are generally transmitted rapidly, and their effects can range from mild colds to life-threatening diseases [40]. These diseases have major social and economic impacts, through both productivity losses caused by work absenteeism and the heavy seasonal burden placed on healthcare systems. Moreover, an emerging virus recently provoked an extreme health emergency, the COVID-19 pandemic, which resulted in at least 700 million cases and seven million deaths worldwide. This pandemic led to a massive effort to rapidly develop affordable vaccines and new antivirals. However, the development of effective new drugs is often hindered by the limitations of traditional screening protocols based on 2D cultures of established cell lines, which may account for the failure of the candidate drugs identified when tested in (pre)clinical assays. Indeed, the cell lines used in these high-throughput screens may not reproduce important conditions relevant to infection progression in human airways [2,4,5,6,37,41]. The need to refine preclinical models is of utmost importance when searching for drugs that target host cell pathways. Since we were searching for drugs targeting virus-induced metabolic adaptations during viral respiratory infections, we used human polarized pseudostratified airway epithelia grown in air-liquid interface. This 3D in vitro model is currently considered the preclinical system that best approximates respiratory diseases in humans [2,6,7,34,35,38,41].

Initially, the effects of the inhibitors were compared in two established cell lines: human MRC5 lung fibroblasts and human Huh7 hepatocarcinoma cells, the latter a line frequently used in antiviral drug screening. While these two cell lines are proliferative and thus should be metabolically programmed to sustain a Warburg phenotype, they are intrinsically different since MRC5 cells are non-transformed fibroblasts, while Huh7 are tumor cells. In these distinct contexts, HCoV-229E behaves differently, exhibiting higher cytolytic activity in Huh7 cells, which required us to adapt our experimental protocols to study the antiviral activity of the inhibitors. Our results indicate that HCoV-229E-induced metabolic adaptations differ between the two cell lines, as they responded distinctly to inhibitors of the PI3K/AKT/mTOR signaling cascade (Figure 2). The differences in the antiviral activity of DCA are particularly striking. These observations would be consistent with the notion that fast-replicating viruses enforce a higher activity of the non-oxidative branch of the pentose phosphate pathway to fulfill the increased demand for nucleotides to synthesize viral DNA/RNA [42]. This would be the case for HCoV-229E in HuH7 cells, while this virus would behave as slow-replicating in a MRC5 scenario, where the potent antiviral activity of DCA would be consistent with a strong inhibition of aerobic glycolysis (lactate formation).

Our work progressed to provide a more physiological context to the investigation of the antiviral activity of DCA against respiratory viruses. In polarized small airway epithelia, the infection with the common-cold coronavirus HCoV-229E progresses as slow-replicating virus and DCA reduced the viral progeny by three orders of magnitude (Figure 6). Similar reductions were observed when epithelia were infected with SARS-CoV-2 or RSV (Figure 7). The effect of DCA was even evident when DCA was added to the basolateral medium at 24 hpi, once infection had already been established. Unfortunately, energy metabolism cannot be characterized in these epithelia in the same way as in MRC5 cells, as the Extracellular Flux Analyzer requires the sample to be immersed in the assay medium. Although the analyzer has already been used with airway epithelia [36,41,43], the physiology will be disturbed by the absence of air and exposure of the apical phase to the assay medium.

The virus-induced reprogramming of cellular metabolism points to glycolytic inhibitors as possible antiviral drugs [12,13,25]. Thus, hexokinase inhibitors such as 2-deoxyglucose (2DOG) or 3-bromopyruvate, as well as the lactate dehydrogenase inhibitor oxamate, have been scrutinized as potential antiviral agents, and notably, 2DOG underwent phase II trials on COVID-19 patients [44]. DCA has been shown to be effective against hepatitis viruses in established tumor cell lines [45,46], Zika virus in cortical progenitor cells from patients [47], West Nile virus infections in the brains of mice [48] and SARS-CoV-2 in isolated human kidney cells [49]. However, DCA has also been shown to enhance the oncolytic activity of adenovirus, measles, or reovirus [50,51]. This suggests that the replication cycle of these latter viruses does not require the preservation of cellular homeostasis. In such a scenario, cellular defense mechanisms will increase the Warburg effect as a rescue pathway against viral-induced cytolytic effects. Consequently, the inhibition by DCA of aerobic glycolysis promotes the oncolytic activity.

Therefore, the available data reinforce the role of aerobic glycolysis in the virus-induced hijacking of cellular metabolism, suggesting that DCA could be a broad-spectrum antiviral particularly for slow-replicating viruses. The beneficial antiviral effects of DCA on airway epithelia are not evident in the prevention of the progression of HCoV-229E infection in Huh7 cells or has even shown to facilitate the oncolytic activity of attenuated measles virus or adenoviruses [50,51]. Therefore, it is essential to emphasize that physiologically relevant disease models must be used to confirm its clinical relevance. Thus, DCA was found to be effective against RSV in small airway epithelia, whereas its antiviral activity in MRC5 cells is weak, and it would probably have been excluded from further study in any screening program.

The biochemical basis of the effects of DCA on cellular energy metabolism is well established. DCA is an inhibitor of PDKs, kinases that regulate the PDH complex (PDC). The PDC is a hub for the Warburg effect and acts as a mitochondrial gatekeeper, controlling the final steps of glucose catabolism. Within the PDC, PDH catalyzes the conversion of pyruvate to acetyl-CoA, thereby linking glycolysis to oxidative phosphorylation. PDKs regulate the activity of PDH by phosphorylation [31]. When phosphorylated, PDH is inactive and lactate dehydrogenase A (LDH-A) converts pyruvate to lactate. The expression of both PDKs and LDH-A is induced by HIF-1α; thus, PDH is considered a drug target not only for cancer treatment but also for other pathologies [52,53,54,55,56].

The physiological activation of PDH can be mimicked by halogenated salts such as DCA through the inhibition of PDK, thereby facilitating the dephosphorylation of PDH [57]. DCA is an investigational drug that was first successfully used in human patients to reduce plasma levels of lactic acid [52] and since then, DCA has been used to treat lactic acidosis, particularly in patients with inherited mitochondrial diseases associated with reduced PDH activity [53,54,55,58], as an antitumour agent [55,59], and in other human pathologies like pulmonary hypertension [56,60] or endometriosis [61,62]. It is important to note that millimolar concentrations of DCA are commonly used in cellular assays because the drug has to mimic the high cellular pyruvate levels, a physiological mechanism that also leads to the inhibition of PDK (Supplemental Table S1). It should be noted that treatment of the epithelia with 10 mM DCA for up to 96 h did not cause alterations in the structure of the epithelia as evidenced by confocal microscopy (Supplemental Figure S4).

Despite the apparently high concentrations used in in vitro assays, this investigational drug has been shown to be effective to treat different human pathologies when administered orally or intravenously twice daily at doses ranging from 5 to 25 mg/kg [54,59,63,64], resulting in peak plasma levels ranging from 0.2 to 1.2 mM [64,65]. Prolonged DCA treatments have been shown to provoke mild secondary effects, which are influenced by age and related to a haplotype of the enzyme glutathione transferase (GSTZ1) involved in DCA clearance and resolve upon drug discontinuation [63,66,67]. These toxicity profiles, associated with prolonged treatments, should be evaluated in the context of antiviral therapies that would generally be used for short periods.

## 4. Materials and Methods

### 4.1. Cell Lines

The human cell lines MRC5 (lung fibroblasts), hSAEC (primary small airway epithelial cells), Huh7 (hepatoma cancer cells) and Hep2 (carcinoma) were obtained from the American Type Culture Collection (Life Technologies, Frederick, MD, USA). The monkey cell line Vero E6 was from Dr. Luis Enjuanes (CNB-CSIC, Madrid, Spain). MRC5 cells were cultured in minimum essential medium (MEM) supplemented with 10% heat-inactivated fetal bovine serum (HIFBS, Gibco, Waltham, MA, USA), 100 U/mL penicillin and 100 μg/mL streptomycin. Vero E6 and Hep2 cells were cultured in Dulbecco’s modified Eagle’s medium (DMEM) supplemented with 10% HIFBS, 100 U/mL penicillin, 100 μg/mL streptomycin and 2 mM L-glutamine. All cells were grown at 37 °C and 5% CO_2_ in a humidified incubator.

### 4.2. Viruses

The recombinant human coronavirus 229E expressing GFP (HCoV-229E-GFP) [68] was kindly provided by Dr. Volker Thiel (University of Zurich, Switzerland). The human coronavirus SARS-CoV-2 strain MAD6 was kindly provided by Dr. Luis Enjuanes (CNB-CSIC, Madrid, Spain), while Omicron was isolated at CBM. Respiratory syncytial virus A expressing the fluorescent protein mKate-2 (RSV-mKate2) was generated as previously described [69]. Viral stocks were prepared in DMEM supplemented with 2% HIFBS for HCoV-229E-GFP and SARS-CoV-2 in DMEM supplemented with 2% HIFBS and 5% DMSO for RSV-mKate2. Viral concentrations were calculated as plaque forming units per ml (pfu/mL) with the exception of RSV-mKate2, which was expressed as fluorescent focus units per ml (ffu/mL). Concentrations were obtained by titration on Vero E6 for SARS-CoV-2 infection, Huh7 cells for HCoV-229E-GFP infection or Hep2 cells for RSV-mKate2 infection.

### 4.3. Differentiation of Small Airway Epithelia in Air-Liquid Interface Cultures

Undifferentiated hSAEC were grown in PneumaCult-Ex Medium (PC-Ex) supplemented with 100 U/mL penicillin and 100 μg/mL streptomycin. The hSAEC cells were differentiated into small airway epithelia according to the STEMCELL Technologies protocols. Undifferentiated hSAEC at passage 1 or 2 were cultured on Costar 12 mm Transwell (0.4 µm pore) PET membrane inserts (STEMCELL Technologies, Cambridge, MA, USA) with 500 µL and 1 mL of PC-Ex medium in the apical and basolateral compartments, respectively. The cells were maintained at 37 °C and 5% CO_2_, and the medium was replaced every 2–3 days. Upon reaching full confluence, the PC-Ex was removed, and the basolateral compartment medium was replaced with PneumaCult-ALI medium (PC-ALI) supplemented with 100 U/mL penicillin and 100 μg/mL streptomycin. The apical compartment was left without medium and thus, the hSAEC cells were exposed to air for at least three weeks at 37 °C in an atmosphere of 5% CO_2_. The basolateral medium was renewed every 2–3 days. This air-liquid interface (ALI) model enabled hSAEC to differentiate into pseudostratified airway epithelia (ALI-hSAEC), and from week two of differentiation onward, any excess mucus in the apical compartment was removed every two days with Dulbecco’s phosphate-buffered saline without Ca^2+^ or Mg^2+^ (D-PBS). The barrier integrity of the differentiated epithelia was assessed by measuring the trans-epithelial electrical resistance (TEER) using a Millicell ERS-2 voltohmmeter (Merck, Darmstadt, Germany) following the protocol described by Stemcell Technologies. For these assays, the PC-ALI in the basolateral compartment was replaced with D-PBS, while the mucus in the apical compartment was washed before adding 600 μL of D-PBS.

### 4.4. Viral Infections

Huh7 and MRC5 cells were seeded in 24-well plates in DMEM (Huh7) or MEM (MRC5) supplemented with 5% HIFBS 24 h prior to infection and incubated at 37 °C and 5% CO_2_. Viral adsorption was carried out in 2% HIFBS for 90 min at 37 °C and 5% CO_2_, with gentle shaking of the plate every 20–30 min. MRC5 cells were infected with HCoV-229E-GFP at a multiplicity of infection (MOI, pfu/cell) of 1, while the MOI for Huh7 was 2. MRC5 cell infection with RSV-mKate2 was achieved at a MOI of 0.05. In all the cases, the volume of adsorption was 120 µL/well and the adsorption medium was then removed to eliminate non-adsorbed viruses, and 300 µL/well fresh MEM with 2% HIFBS was added to each well.

To study energy metabolism, MRC5 cells were infected on XFp microplates. 5 × 10^3^ cells were seeded in MEM supplemented with 5% HIFBS at 20–30% confluence, and on the following day the medium was removed, and 9 µL/well of 2 × 10^7^ pfu/mL HCoV-229E-GFP were added. After 90 min of adsorption, an additional volume of 25 µL of MEM supplemented with 2% HIFBS was added to each well, and measurements were performed at 48 hpi.

ALI-hSAEC were infected with HCoV-229E-GFP, SARS-CoV-2 or RSV-mKate2 in the apical compartment. After the epithelia were washed three times with prewarmed D-PBS to remove excess mucus, HCoV-229E-GFP (5 × 10^3^ pfu/mL) or RSV-mKate2 (5 × 10^2^ pfu/mL) was inoculated apically in 100 µL D-PBS and incubated for 90 min at 37 °C and 5% CO_2_. SARS-CoV-2 (5 × 10^4^ pfu/mL) was inoculated in 200 µL of D-PBS and incubated for 2 h. Subsequently, the inserts were apically rinsed three times with pre-warmed D-PBS to remove non-adsorbed viruses and left exposed to air, while the PC-ALI medium in the basolateral compartment was replaced with fresh medium. ALI-hSAEC infections were incubated at 37 °C and 5% CO_2_ for up to 72 h, changing the basolateral medium after 48 h. All the experiments involving SARS-CoV-2 were performed in the biosafety level 3 containment laboratory at CBM (CSIC-UAM, Madrid, Spain).

### 4.5. Quantification of Viral Infection

Huh7 and MRC5 cultures were trypsinized (trypsin-EDTA 0.05%), washed with phosphate-buffered saline (PBS) and subsequently fixed with 4% paraformaldehyde (PFA) for 12 min. After removing PFA, the cells were resuspended in PBS, and virus fluorescence was evaluated by flow cytometry in a CytoFLEX S cytometer (Beckman Coulter, Indianapolis, IN, USA). Infection of viruses expressing fluorescent proteins was assessed with excitation and emission wavelengths of 488 and 525 nm, respectively, for HCoV-229E-GFP and of 561 nm and 610 nm for RSV-mKate2. HCoV-229E-GFP infection in ALI-hSAEC was evaluated using a Leica AF6000 LX widefield multidimensional microscopy system (Leica Microsystems, Wetzlar, Germany). Virus-driven GFP fluorescence was quantified with ImageJ/Fiji software, version 2.9.0/1.53c.

The release of newly generated viruses from within the epithelia at the external apical surface was evaluated by plaque assays for HCoV-229E-GFP and SARS-CoV-2, and by quantifying the fluorescent foci for RSV-mKate2. The externalized viruses were collected by apical washing with tempered 200 µL of D-PBS at 37 °C for 10 min, which was added to an equal volume MEM supplemented with 2% HIFBS for HCoV-229E-GFP, and DMEM supplemented with 2% HIFBS for SARS-CoV-2. The samples were stored at −70 °C until they were titrated by plaque assay in Huh7 cells (HCoV-229E-GFP), Vero E6 cells (SARS-CoV-2) or Hep2 cells (RSV-mKate2). Cell monolayers in 12-well plates were inoculated with 10-fold serial dilutions of the medium containing the virus and incubated for 1 h at 37 °C with shaking every 15–30 min. After removing the virus inoculum, HCoV-229E-GFP-infected Huh7 cells were overlaid with medium containing 0.7% agarose and 90 µg/mL diethylaminoethyl-dextran (DEAE-dextran), and the plates were incubated for 4 days at 33 °C. In the case of SARS-CoV-2, Vero E6 cells were overlaid with semisolid medium containing 1.5% carboxymethylcellulose and 2% HIFBS, and the plates were incubated for 3 days at 37 °C. In all the cases, the cells were then fixed in 4% or 10% formaldehyde for at least 30 min at room temperature (RT) and stained with 3% crystal violet in 2% formaldehyde. Finally, the plates were washed, and the virus-induced plaque formation was quantified. RSV infectious viral titres were determined by quantifying the fluorescent foci. Briefly, Hep2 monolayers in 96-well plates were inoculated with 100 µL of 10-fold serial dilutions of the virus-containing media and incubated for 24 h at 37 °C. The fluorescent foci from 4 replicates inoculated at each dilution were identified and quantified by fluorescent microscopy (DM fluorescence inverted microscope, Leica Microsystems, Wetzlar, Germany) using a Texas Red filter (Leica Microsystems, Wetzlar, Germany).

### 4.6. Energy Metabolism

MRC5 and Huh7 cell bioenergetics were characterized using either an XFe24 or an XFp Extracellular Flux Analyzer (Agilent Technologies, Santa Clara, CA, USA), which enabled the determination oxygen consumption rate (OCR) and the rate of aerobic glycolysis (derived from the extracellular acidification rate, ECAR). The ECAR values were corrected to take into account the CO_2_ contribution, as described previously [70]. MRC5 and Huh7 cells were seeded in XFe24-well or XFp microplates (Agilent Technologies) in MEM and DMEM, respectively. One hour prior to the OCR and ECAR measurements, the culture medium was carefully removed, the wells were washed with assay medium (bicarbonate-free DMEM containing HEPES with 2% HIFBS, 2 mM glutamine, 5 mM glucose, pH 7.4, and, finally, 500 µL (XFe24) or 200 µL (XFp) of assay medium was added. The cells were then maintained for 1 h at 37 °C in a CO_2_-free incubator before the experiment commenced. Experiments with virus-infected MRC5 cells were carried out in the same medium but in the absence of HIFBS. At the end of the experiment, the wells were washed with prewarmed PBS, the cells were lysed with CelLytic-M, and the protein concentration was determined using the bicinchoninic acid assay and bovine serum albumin as a standard.

### 4.7. Confocal Microscopy

Differentiated epithelia were flat-mounted on nitrocellulose membranes, and blocked for 2 h at RT with 10% BSA (*w*/*v*) and 0.1% Triton-X100 (*w*/*v*) in PBS. F-actin filaments were stained overnight at 4 °C with phalloidin–Alexa Fluor 488 in 2% BSA (*w*/*v*) and 0.1% Triton-X100 (*w*/*v*) in PBS, together with a mouse anti-α-tubulin antibody and anti-α-MUC5AC/917-Alexa Fluor 594. After rinsing in PBS, samples were incubated for 2 h at RT in 5% BSA (*w*/*v*) and 0.1% Triton-X100 (*w*/*v*) in PBS with a goat anti-mouse Alexa Fluor 647 antibody and DAPI for nuclear staining. After rinsing in PBS, the samples were cover-slipped with Fluoroshield^TM^ and imaged on a Leica TCS SP8 confocal microscope using a 63× objective. Optical sections were acquired at 1 µm (xyz) and 0.5 µm (xzy). Three-dimensional reconstructions were generated with LAS X Office software, version 1.4.7 (Leica Microsystems). Negative controls included epithelia processed in parallel without primary antibody or phalloidin.

### 4.8. Reagents and Media

PC-Ex and PC-ALI medium, and D-PBS were obtained from Stemcell Technologies (Vancouver, BC, Canada). MEM, DMEM, HIFBS, penicillin/streptomycin, trypsin-EDTA and L-glutamine were all purchased from Gibco (Thermo Fisher Scientific, Boston, MA, USA). Phalloidin and the goat anti-mouse Alexa Fluor 488 antibody were from Thermo Fisher Scientific (Waltham, MA, USA), anti-α-MUC5AC/917 Alexa Fluor 594-conjugated from Novus Biologicals (Denver, CO, USA) and the anti-α-tubulin antibody and DAPI were supplied by Merck. All other reagents and chemicals were obtained from Merck (Sigma-Aldrich, St. Louis, MO, USA) unless otherwise stated.

### 4.9. Statistical Analysis

All the values are expressed as the mean ± s.e.m. The differences between groups were determined using either two-tailed unpaired Student’s *t* tests or one-way ANOVA test using SigmaPlot software (version 11, Jandel Scientific, Leighton Buzzard, UK). Significant differences between groups are indicated as * *p* < 0.05, ** *p* < 0.01 or *** *p* < 0.001.

## 5. Conclusions

Our work further supports aerobic glycolysis, a cellular metabolic pathway commonly hijacked by slow-replicating viruses, as a viable therapeutic target for diverse respiratory viruses. Targeting host–cell metabolic pathways not only reduces the likelihood of antiviral resistance but also allows for potential synergistic effects with drugs aimed at intrinsic viral targets. Our findings also highlight the strategic value of drug repositioning, which can substantially reduce both time and financial resources required to bring a molecule to market, as safety and preclinical data are already available. The experimental setting used, human small airway epithelia in air-liquid interface, provides high physiological relevance to the results demonstrating the antiviral efficacy of DCA against three respiratory viruses that have major impacts on human health including SARS-CoV-2 and RSV.

## Data Availability

The original contributions presented in this study are included in the article and Supplementary Materials. Further inquiries can be directed to the corresponding authors.

## Supplementary Materials

The following supporting information can be downloaded at: https://www.mdpi.com/article/10.3390/ijms26209853/s1. Referencens [31,57,71,72,73,74,75,76,77] are cited in Supplementary Materials.

## Abbreviations

The following abbreviations are used in this manuscript:

ALI	Air-liquid interface
DAPI	4′,6-diamidino-2-phenylindole
DCA	Dichloroacetate
DMEM	Dulbecco’s modified Eagle’s medium
D-PBS	Dulbecco’s phosphate-buffered saline
ECAR	Extracellular acidification rate
EGFR	Epidermal growth factor receptor
EVL	Everolimus
ffu	Fluorescent focus units
HER2	Human epidermal growth factor receptor 2
HIF-1α	Hypoxia inducible factor 1α
HIFBS	Heat-inactivated fetal bovine serum
hpi	Hours post-infection
hSAEC	Primary human small airway epithelial cells
LDH-A	Lactate dehydrogenase A
LPT	Lapatinib
mTOR	Mammalian target of rapamycin
OCR	Oxygen consumption rate
MEM	Minimal essential medium
MOI	Multiplicity of infection
PBS	Phosphate-buffered saline
PDC	Pyruvate dehydrogenase complex
PDH	Pyruvate dehydrogenase
PDK	Pyruvate dehydrogenase kinase
PFA	Paraformaldehyde
PI3K	Growth factor-regulated phosphoinositide 3-kinase
pfu	Plaque forming units
RSV	Respiratory syncytial virus
RT	Room temperature
TEER	Transepithelial electrical resistance
WTM	Wortmaninn
2DOG	2-deoxyglucose
2ME	2-methoxyestradiol
