# Supplementary material for: Human Small Airway Epithelia Reveal Dichloroacetate as a Broad-Spectrum Antiviral Against Respiratory Viruses
Source: Int J Mol Sci. 2025 Oct 10;26(20):9853. doi: 10.3390/ijms26209853 (PMC12563306; doi:10.3390/ijms26209853)
Supplement: Supplementary file 1 [file ijms-26-09853-s001.zip › Supplemental Figures S1-S4.pdf]

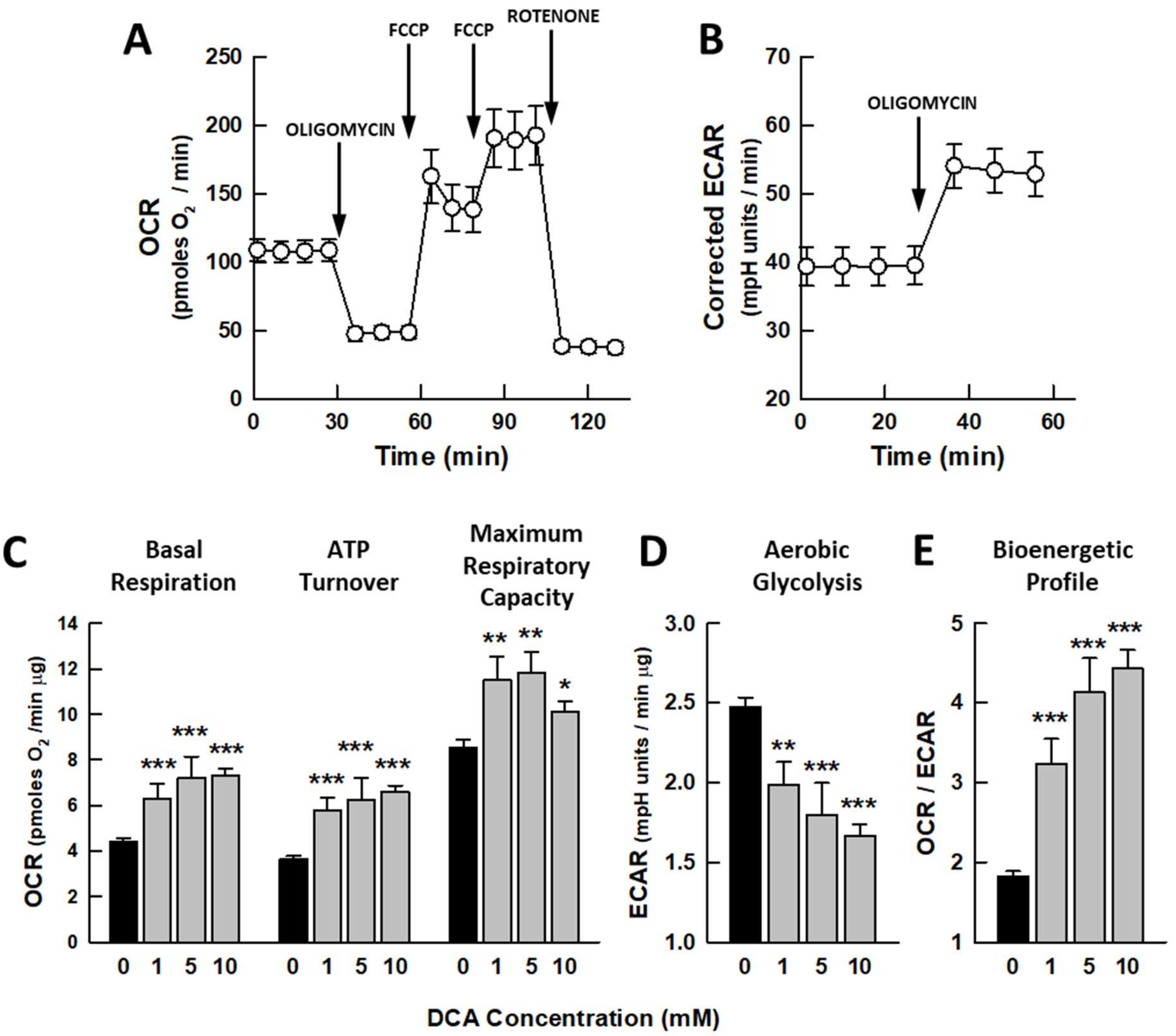

Figure S1. DCA alters the energy metabolism of the cell line MRC5. (**A**-**B**) Bioenergetic profiling of MRC5 cells using the mitochondrial stress test. (**A**) Average profile of the changes in cellular respiration (OCR) following sequential addition of oligomycin (1 μM) followed by the two additions of the uncoupler FCCP (0.6 and 0.4 μM), and rotenone (1 μM) plus antimycin A (1 μM). (**B**) Changes in aerobic glycolysis (ECAR changes corrected to subtract the CO_2 contribution) and the effect of oligomycin (1 μM). (C–E) Effect of DCA on bioenergetic parameters derived from the assay in (**A**–**B**): (**C**) basal respiration, ATP turnover, and maximal respiratory capacity; (**D**) basal aerobic glycolysis; (**E**) OCR/ECAR ratio. Data in (**A**–**B**) represent means ± s.e.m. of 14 independent experiments; bar graphs in (**C**–**E**) represent means ± s.e.m. of 4–5 independent experiments. Statistical significance was assessed by one-way ANOVA: *P < 0.05, **P < 0.01, ***P < 0.001.

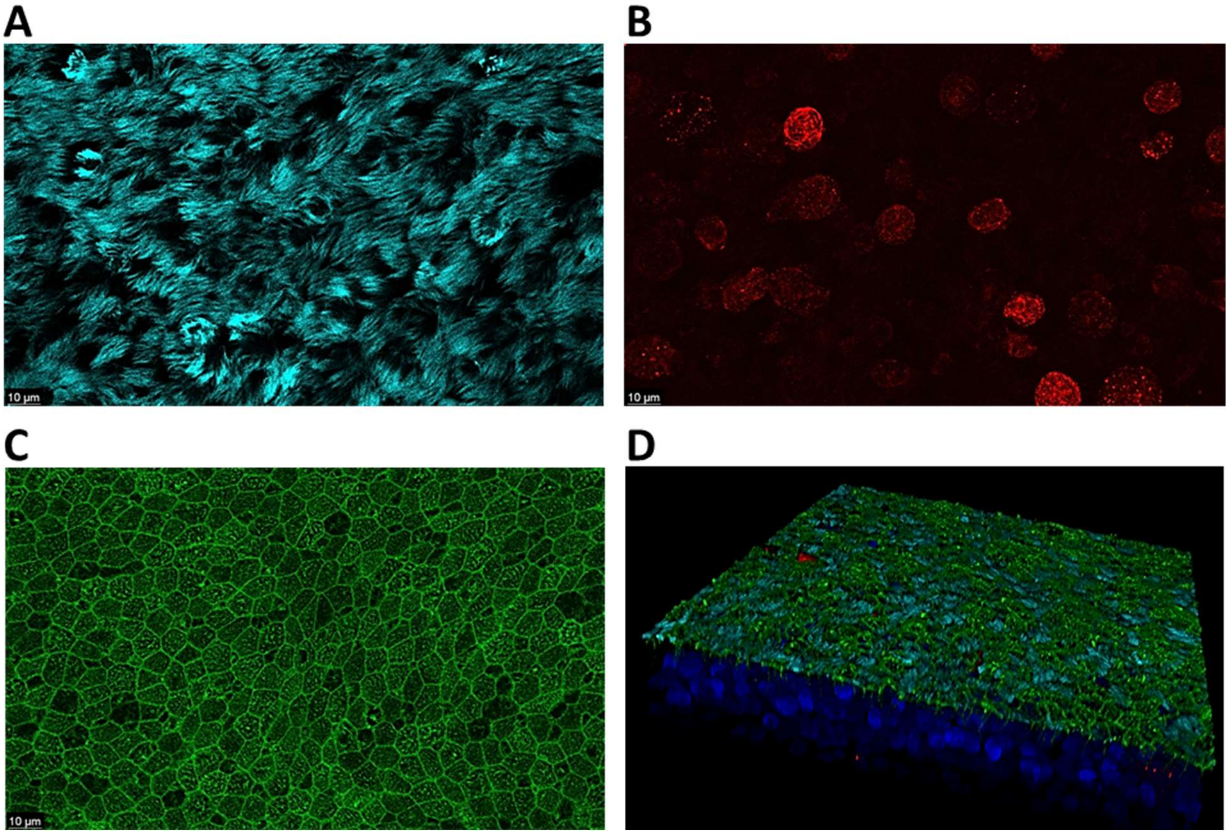

Figure S2. Representative images of the characterization by confocal microscopy of the human small airway pseudo-stratified epithelia used in the studies. (**A**) Top-view visualization of cilia by α-tubulin staining. (**B**) Detection of goblet cells by MUC5AC immunostaining. (**C**) Visualization of cell borders in the apical epithelial layer by F-actin staining. (**D**) Representative 3D reconstruction of the pseudo-stratified epithelium showing α-tubulin (cilia, cyan), F-actin (green), MUC5AC (red), and nuclei (DAPI, blue).

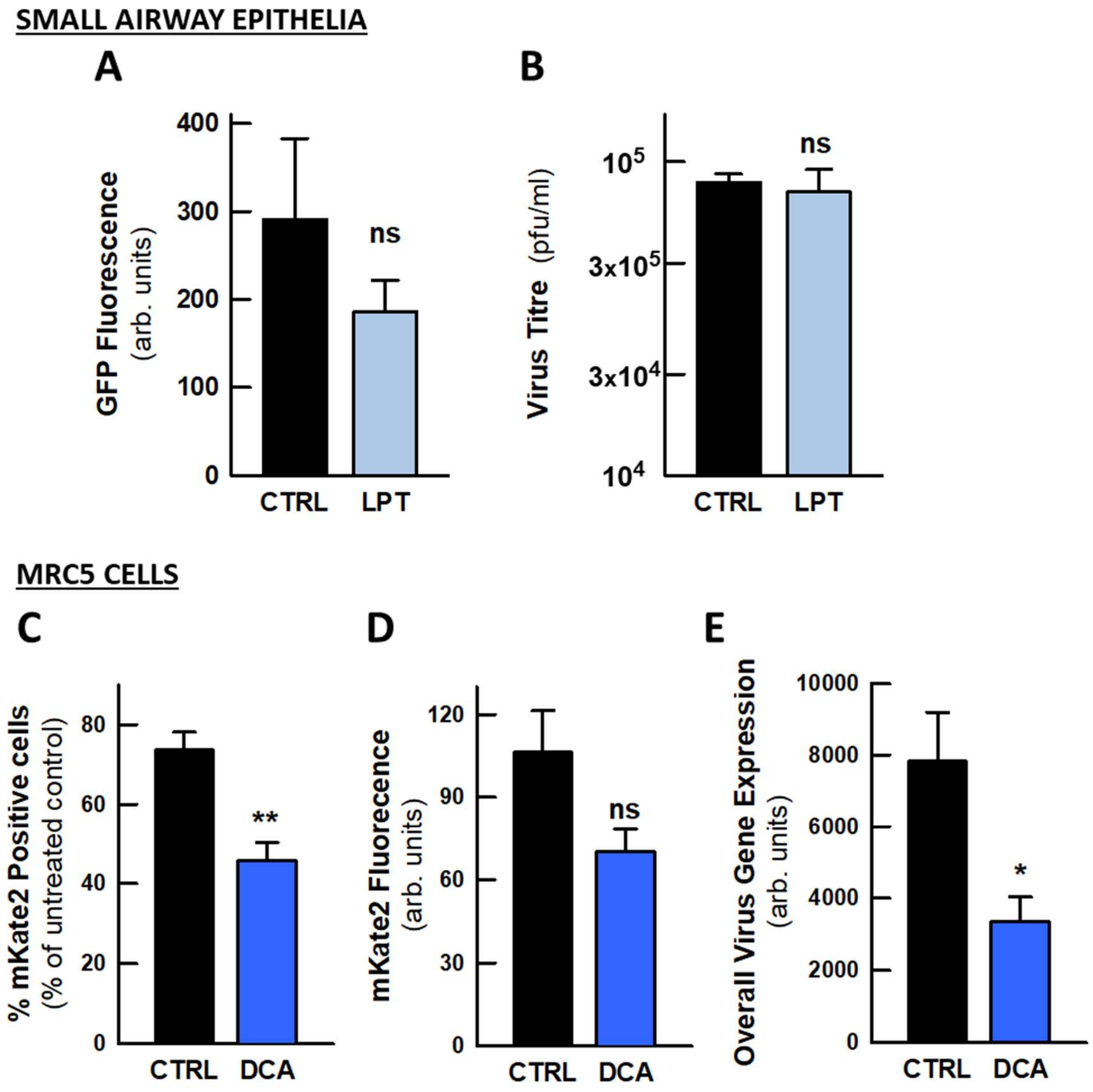

Figure S3. Weak antiviral activity of LPT in airway epithelia infected with the coronavirus HCoV-229E and of DCA in MRC5 cells infected with the pneumovirus RSV. (**A**–**B**) Effect of LPT (5 µM) in airway epithelia infected with HCoV-229E: (**A**) GFP fluorescence at 72 hpi and (**B**) viral titers in apically recovered mucus at 72 hpi. (**C**–**E**) Effect of DCA (10 mM) in MRC5 cells infected with RSV: (**C**) percentage of infected cells, (**D**) mean GFP fluorescence of infected cells, and (**E**) overall viral gene expression (calculated from the percentage of infected cells and the mean fluorescence intensity). Data are presented as means ± s.e.m. In (**A**), 12 inserts per condition were analyzed; in (**B**), 12 control and 7 LPT-treated inserts; in (**C**–**E**), 8 control and 5 DCA biological independent experiments. Statistical significance was determined by unpaired Student's *t*-test: $^*P < 0.05$, $^{**}P < 0.01$, n.s., not significant difference.

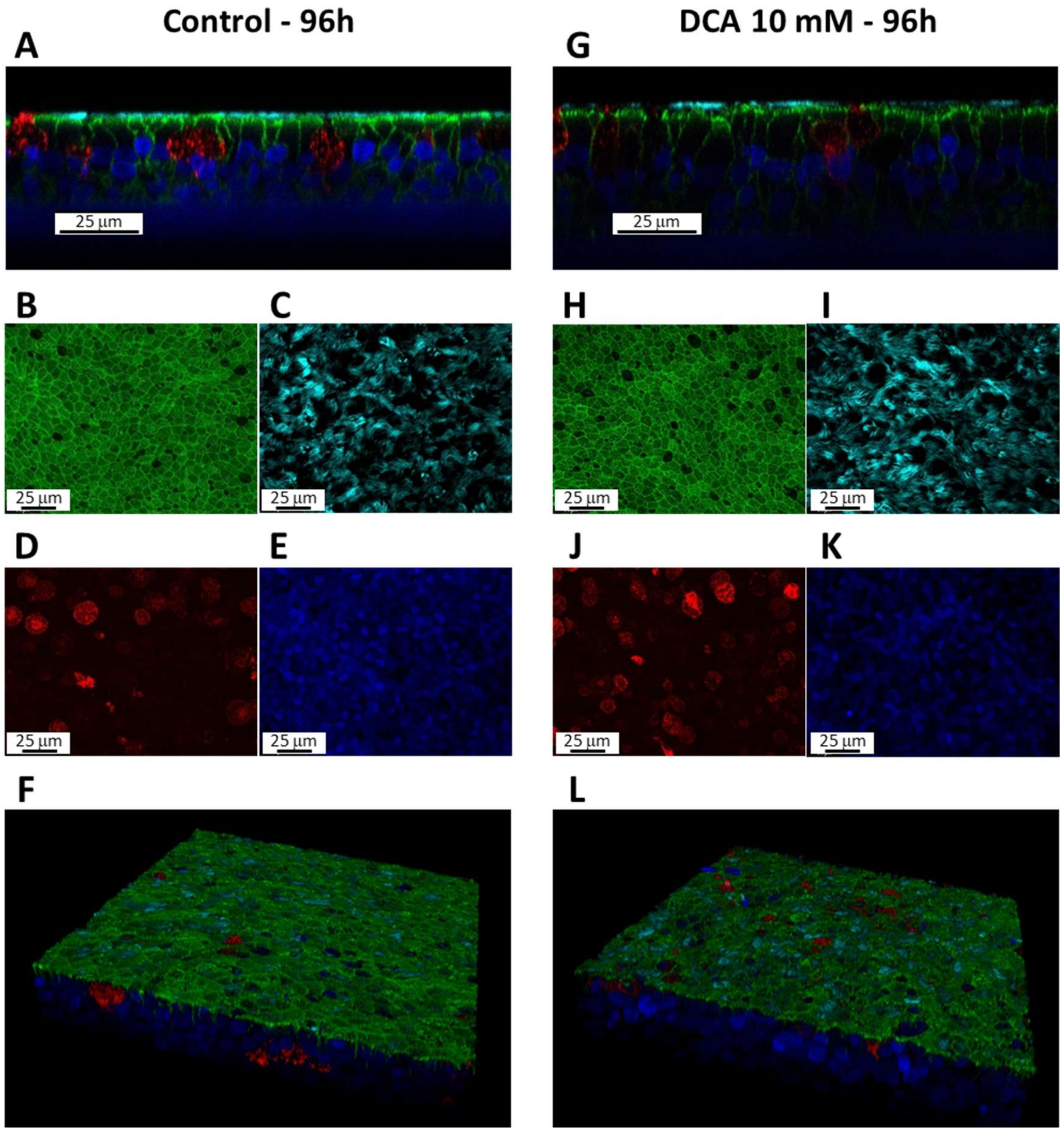

Figure S4. Confocal microscopy of small airway epithelia after 96 h treatment with DCA. (**A**-**F**) Control epithelia; (**G**-**L**) epithelia treated with 10 mM DCA applied to the basolateral compartment for 96 h. Basolateral medium was refreshed every 2 days when excess apical mucus was removed by washing with D-PBS. Staining as described in Figure S2: nuclei (blue), F-actin (green), α-tubulin (cyan), and MUC5AC (red). (**A**, **G**) Side views of the pseudostratified epithelia; (**B**–**E**, **H**–**K**) apical views; (**F**, **L**) 3D reconstructions.
