# Supplementary material for: Human Small Airway Epithelia Reveal Dichloroacetate as a Broad-Spectrum Antiviral Against Respiratory Viruses
Source: Int J Mol Sci. 2025 Oct 10;26(20):9853. doi: 10.3390/ijms26209853 (PMC12563306; doi:10.3390/ijms26209853)
Supplement: Supplementary file 1 [file ijms-26-09853-s001.zip › Supplemental Table S1.pdf]

Supplemental Table 1. Reference values for inhibitors targeting pathways associated with the Warburg effect. Reported IC_{50} values were obtained from studies employing either purified target assays (enzyme) or cell-based assays measuring effects on cell proliferation. For each inhibitor, the concentration ranges most frequently reported in the literature are provided, together with the concentrations used in the present study with MRC5 and Huh7 cell lines.

		Reported IC_{50}		Concentration ranges commonly used	Concentrations tested		References
Inhibitor*	Target	Enzyme	Cancer cell growth		MRC5	Huh7	
Lapatinib	HER2 / EGFR	0.1 – 10 μM	50 nM – 12 μM	0.1 – 20 μM	1 & 5 μM	2 & 5 μM	[71,72]
Wortmannin	PI3K	2 nM	400 nM	1 nM – 1 μM	10 nM & 1 μM	10 nM & 1 μM	[73,74]
Everolimus	mTORC1	1 – 2 nM	20 – 40 nM	1 – 100 nM	10 & 100 nM	10 & 100 nM	[75,76]
2-Methoxyestradiol	HIF-1α	N/A	0.5 – 3 μM	0.1 – 10 μM	2 μM	n.d.	[77]
Dichloroacetate	PDK	0.5 – 1.5 mM	10 – 50 mM	0.5 – 50 mM	1, 5 & 10 mM	5 & 10 mM	[31,57]

*Links to the NIH-PubChem database provided

Abbreviations:

IC_{50}, Half-maximal inhibitory concentration; HER2, Human epidermal growth factor receptor 2; EGFR, Epidermal growth factor receptor; PI3K, Phosphoinositide 3-kinase; mTORC1, Mammalian target of rapamycin complex 1; HIF-1α, hypoxia-inducible factor 1α; PDK, Pyruvate dehydrogenase kinase; N/A, non-applicable; n.d., not determined.
